# Raising enthusiasm for the medical care of elderly patients: a concept mapping study to find elements for an elderly friendly medical curriculum

**DOI:** 10.1186/s12909-018-1344-6

**Published:** 2018-10-20

**Authors:** Ariadne A. Meiboom, Henk de Vries, Fedde Scheele, Cees M. P. M. Hertogh

**Affiliations:** 1 Department of General Practice & Elderly Care Medicine, VU University Medical Center, Van der Boechorststraat 7, 1081 BT Amsterdam, The Netherlands; 2 Department of Research in Education, VU University Medical Center, Van der Boechorststraat 7, 1081 BT Amsterdam, The Netherlands

**Keywords:** Medical students, Geriatrics, Curriculum development, Concept mapping

## Abstract

**Background:**

To deliver high quality of care for the growing population of older patients more geriatricians are needed. However, the interest of medical students for a career in geriatrics is lagging behind due to a lack of exposure, the nature of the work, and the low status and financial rewards.

So far, only isolated interventions aimed at enhancing interest and/or attitudes with regard to geriatrics have been studied, pointing to the need for a broader-based strategy. The goal of this research is to find elements for a curriculum framework that can raise medical students’ enthusiasm for the medical care of elderly patients.

**Methods:**

We used the concept mapping method developed by Trochim. This computer-assisted procedure consists of five steps: brainstorming, prioritizing and clustering with several experts, followed by processing by the computer and analysis.

**Results:**

The views that were generated were grouped into the following clusters: a patient-centered medical curriculum, a curriculum representative of patient population, geriatrics presented as intellectually challenging and emotionally appealing, senior-friendly role models, a clear professional perspective.

The results are presented in the form of a graphic chart.

**Conclusions:**

An agenda to discuss the necessary actions for drastic curricular reforms in medical schools is set. This may give some guidance to this urgent, but highly complicated issue how to make medical student enthusiastic for the medical care for elderly patients.

## Background

There is an increasing demand for geriatricians or physician workforce to care for older people, due to a growing population of elderly people. For instance, in the Netherlands the demand for geriatricians is estimated to increase by 30% over the next 10 years [1]. In the United States geriatricians are in dangerously short supply [2]. A review that summarizes studies that assess the quality of care of vulnerable elderly in different settings, concludes that the quality of care for elderly people is poor [3]. The care for geriatric conditions showed greater deficiencies than the care for general conditions. To improve the quality of care for older patients with general conditions, more geriatricians are needed to train all medical students and residents, to conduct research and to develop standards of care for vulnerable older patients [4]. In addition, according to American academic geriatric medicine leaders, all vulnerable patients aged 85 and older with geriatric syndromes and functional impairment should have geriatrician’s care [4, 5].

In Europe healthcare systems differ among countries. In some countries geriatric care can also be delivered by nursing home physicians or by general practitioners and internists. In general, there is a need for a growing physician workforce caring for older people in Europe and medical students have to be trained in the skills of geriatric medicine [6, 7].

However, there is very little interest among medical students for a career in geriatrics [8, 9], or to work with the elderly [10], due to a lack of exposure to this discipline (limited geriatric didactic content and especially limited geriatric clinical experiences), the nature of the work (chronic diseases and the complexity of geriatric patients) and the profession’s low status and financial rewards [11]. Besides, even though a considerable proportion of hospital admissions concerns elderly patients [12], residents and medical students show mixed attitudes regarding the care for elderly patients, among which frustration with the hospital system, which seems to place more value on short-term efficiency and cure instead of (personalized) care [13, 14].

A systematic review reporting on the effect of educational interventions on undergraduate knowledge, skills and attitudes in geriatric medicine showed a mixed picture regarding attitudes, although interventions of longer duration (years rather than hours or days) were more likely to improve attitudes than brief interventions [15]. In the United States, several medical schools adopted Senior Mentor programs to reduce stereotypes about aging. In these programs medical students are matched with independent, relatively healthy older adults for a period of time. According to a national evaluation of 10 senior mentor programs, all demonstrated a positive effect on student attitudes towards older adults [16].

Several medical schools adopted more integrated geriatric content in the curriculum. One medical school with geriatrics integrated in problem-based learning and standardized patients throughout the first three years and a required fourth year rotation in geriatrics showed positive effects on student self-efficacy, knowledge and skills. However, attitudes did not change significantly [17].

As the reliability and validity of the measures commonly used to assess medical students attitudes are questionable [18], our research group in a previous study applied medical students’ interest in geriatrics as an outcome measure in relation to different factors [11]. That search yielded several studies of medical students’ interest in relation to curricular interventions.

Regarding preclinical education, one study reported that medical students who participated in an extracurricular program that partnered medical students with community-dwelling elders, had a significantly higher likelihood of being interested in geriatric medicine at the end of medical school [19]. Two other studies, however, found no significant increase in interest in geriatrics among medical students after a preclinical geriatric course [20, 21].

With regard to clinical education, two studies reported a significant increase in interest in a career in geriatrics after a clinical attachment in this field. Two other studies also reported an increase in interest after a geriatrics attachment but did not mention the statistical significance. However, a longitudinal study showed that interest in geriatrics decreased between the completion of a fourth year attachment in health care of the elderly and graduation [22].

Despite promising innovations in medical schools, such as senior mentor programs and clinical attachments, there are still not enough medical students interested in a career in geriatrics. Given the urgency of the situation, we want to advocate a more comprehensive approach. The main aim of this research therefore is to find elements for a curriculum framework, which might raise medical students’ enthusiasm for the medical care of elderly patients.

We will explore through concept mapping which guiding themes are important in the medical curriculum to raise the interest of the students for the medical care of elderly patients, to set an agenda for further discussion.

## Methods

Concept mapping, as developed by Trochim, is a particularly suitable method for mapping complex, not yet fully crystallized topics, into underlying concepts [23]. It is designed to integrate input from experts with different backgrounds, by producing an interpretable visual map of their ideas and concepts and how these might be interrelated. Due to its structured format, which also includes free individual association on the topic, concept mapping is more resistant than focus groups to the effects of group dynamics. It consists of five steps: brainstorming, prioritizing, clustering, processing by the computer and analysis (multivariate statistical methods of multidimensional scaling and hierarchical cluster analysis), resulting in a visual map. By interpreting the clusters and their thematic differences, and naming the clusters and the axes, the map results in a conceptual framework.

It has been demonstrated that the internal representational validity of concept mapping is strong and the sorting and rating reliability are very strong [24]. In the Netherlands the use of concept mapping is well established. It has been used, for example, to explore different topics in healthcare, such as coping with illness [25], and using surveillance technology in residential care [26], and it turned out to be a suitable method for exploring difficult subjects with different people over a relatively short period of time.

### Participants

The research group invited three categories of experts: medical students, curriculum designers and physicians. To find enough participants, the research group selected 10 professionals from different medical schools - although the eight medical schools in the Netherlands do not differ substantially - with extensive experience in (re)building medical school curricula, among which professionals with a position as an associate Dean, and 10 physicians who were active in patient care with older patients and active in medical education. Moreover, the research group asked students from the student panel that advises the Dean on improving the curriculum to participate. Of the invited participants, eight were able to attend the concept mapping session, i.e. three curriculum designers and three physicians, including one geriatrician, one elderly care physician, and one resident, and two medical students. The three curriculum designers are all experienced in (re)building medical school. Of the curriculum designers who did not attend, two have the same profile and three were associate deans. Written consent was obtained from the participants.

### Procedure

The concept mapping session took place on March 11, 2014, under the supervision of an independent chair specialized in working with the concept mapping method. The session entailed the following:

Step one (brainstorming): The session started with brainstorming in which the participants were asked to complete the seeding statement, prepared by the research group:

“ A medical curriculum can only raise the enthusiasm of medical students for the medical care of elderly patients if….”

This task had to be carried out individually. When all participants had completed this task, they presented their statements in a group session. They were allowed to engage in discussion, but only to clarify the statements. The chair collected all statements on the computer.

Step two (prioritizing): The participants were then asked to rate the importance of all collected statements by dividing them into five groups of equal size, thus preventing all statements being valued the same or too high. Statements in group 1 were considered to be least important and statements in group 5 most important. This task was also carried out individually.

Step three (clustering): The participants were also asked to cluster the statements individually into groups of common features. A statement could only be used once. The number of groups was limited to ten by the software.

The subsequent steps were completed by the researchers, without the involvement of the participants.

Step four (analysis): Aided by the software program Ariadne [27] two types of analyses were conducted. First, the statements were positioned in a two-dimensional concept map, based on the clustering results. The location of each statement matters, as the distance between the statements represents how often these statements are placed together in a group by the participants. Second, the individual statements with close proximity on the map were grouped into clusters of statements that reflect similar concepts. The individual statements were joined in clusters of interrelatedness through a process called hierarchical cluster analysis. The number of clusters was determined by the researchers, by looking at all cluster solutions grouped by the computer, examining which statements were grouped together in a cluster and deciding whether this grouping made sense.

The importance of each cluster was calculated based on the average score of the importance awarded to each statement by the participants in step two.

Step five (interpretation): In this phase of interpretation the researchers discussed the significance of the clusters and their thematic differences and finally named the clusters and the axes.

The research group consisted of two elderly care physicians (CH and AM), one general practitioner (HdV) and one gynaecologist (FS), all with a special interest in medical education.

According to Dutch law this type of research requires no ethical review [28]. In addition, the medical students that participated in our panel were consulted not in the context of their study program, but in their capacity as members of a student panel that advises the Dean on improving the curriculum.

## Results

### Statements

**Fig. 1:**
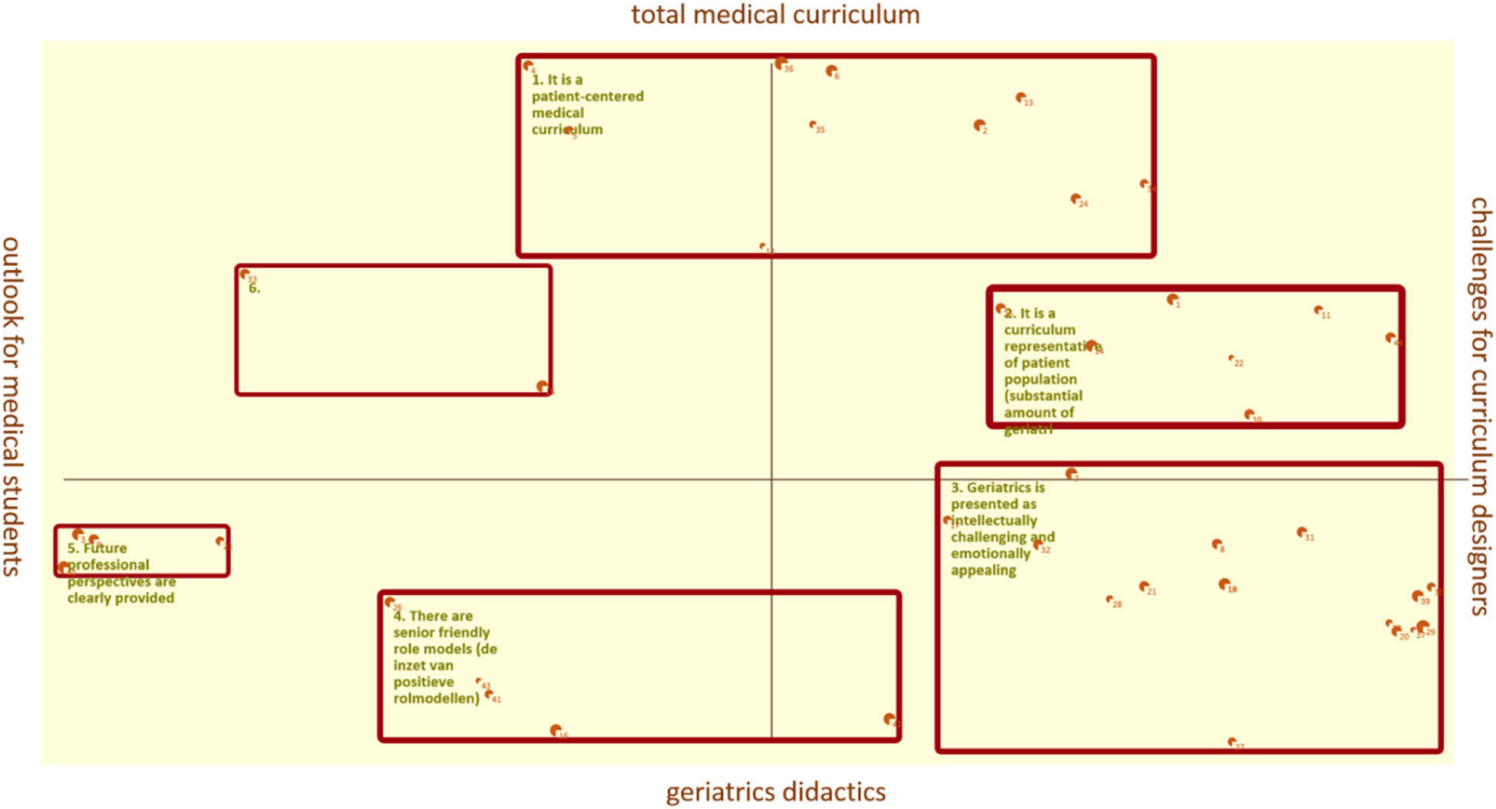
Concept map. Each box is a cluster. The thickness of the red line represents the average rating for that cluster. The thickness of each point represents the average rating for that statement

The seeding statement yielded 44 statements by the participants. All statements are listed in Table 1. A top ten of statements is listed in Table 2. The concept map is shown in Fig. 1.

**Table 1:** Statements

Statement number	Statement
Cluster 1. It is a patient centered medical curriculum	
2	Attention for the whole patient is presented as a challenge
4	Give insight into the limited curative ability of medicine
5	Insight into preventive possibilities
6	From cure to care
12	Insight into the patient population in the hospital
13	Less profession specific and more towards patient problems
24	Comorbidity instead of one single diagnosis
34	Think about social responsibility within the curriculum
35	Less emphasis on the biomedical paradigm
36	Start with patient perspective instead of the biomedical
Cluster 2. It is a curriculum representative of patient population (substantial amount of geriatrics).	
1	A balanced curriculum, which is representative for medicine
10	Continuous exposure to geriatrics throughout the whole curriculum
11	70% of medical cases for medical students should consist of elderly patients
14	Elderly patients are not boring or difficult
22	Assessment of geriatric content
25	Death and dying should have a central space in the curriculum
44	Interdisciplinary education
Cluster 3. Geriatrics is presented as intellectually challenging and emotionally appealing.	
7	The visibility of the scientific challenge of aging
8	The scientific gap that is there to discover is a great challenge
17	Promotion of the field by prestigious individuals and by medical specialists
18	The involvement of own family and loved ones within the curriculum
19	Creating empathy by more exposure to older people within the curriculum
20	Making use of current affairs or spectacular topics
21	Bringing the message that geriatrics is exclusive and for the very talented
27	The geriatric clerkship should be the best
28	An award for the most talented student regarding geriatrics
29	Organizing attractive elements in geriatrics such as e-health or games
30	Geriatric literature and curriculum have to be of high quality
31	Intellectually challenging and emotionally appealing
32	Elderly people as a role model
37	Making use of reports in the media in the curriculum
38	Organization of journal clubs
39	A separate compulsory geriatric clerkship
Cluster 4. There are senior friendly role models.	
16	Role models in geriatrics
26	Early attention to professional identity development during medical school
41	Acknowledging that some physicians are not elderly minded
42	Proud teachers
43	Teacher professionalization on the job
Cluster 5. Future professional perspectives are clearly provided.	
3	It must be clear to medical students who the key players in the field are
9	Insight into career perspectives
23	Insight into career perspectives including financial rewards
40	Helping students in gaining insight into what kind of doctor they want to become
Cluster 6.	
15	Making clear wich treatment options there are
33	From individual physician to team player(s)

All statements generated by the participants, sorted through hierarchical cluster analysis

**Table 2:** The 10 most important statements of the concept mapping session

Statement number	Statement	Mean importance
10	continuous exposure to geriatrics throughout the whole curriculum	4.5
1	a balanced curriculum, which is representative for medicine	4.38
4	give insight in the limited curative ability of medicine	4.38
17	promotion of the field by prestigious individuals and by medical specialists	4.38
16	role models in geriatrics	4.25
25	death and dying should have a central place in the curriculum	4.25
39	a separate compulsory geriatric clerkship	4.14
21	geriatrics is exclusive and for the very talented	4.0
2	attention for the whole patient is represented as challenging	3.75
6	from cure to care / searching for the scientific challenge of aging	3.63

Mean statement importance (sorted)

### Clusters

Based on the sorting of the 44 statements, six clusters were created during the fourth step of the concept mapping process. These clusters are described below. The numbers in parentheses, varying from 1 to 5, represent the mean importance of the statements in that cluster. A higher number means higher importance.

The numbering of the clusters represents the clockwise sequence on the concept map (see also Fig. 1).

Cluster 1: It is a patient-centered medical curriculum (mean importance 3.14).

This cluster contains ten statements, three of which can be found in the top ten.

The emphasis in this cluster is on issues that, although characteristic for geriatrics, should be more accentuated in the whole field of medicine and the total medical curriculum. This is illustrated by the three top ten statements of this cluster: “attention for the whole patient is represented as challenging”, “giving insight into limited curative ability of medicine” and “from cure to care”.

As of Cluster 2: It is a curriculum which is representative of the patient population (mean importance 3.5).

Three of the total of seven statements in this cluster can be found in the top ten. This cluster indicates the importance of a proportional amount of geriatric content and attention for the elderly patient integrated within the whole medical curriculum, as represented by statements like “continuous exposure to geriatrics throughout the whole curriculum” and “70% of medical cases for medical students should consist of elderly patients”. The participants explained the statement “a balanced curriculum, which is representative of the field of medicine” as follows: while most clinical encounters involve elderly or chronically ill patients, the management of chronic conditions is an important task in medicine that needs much more attention in medical school.

Cluster 3: Geriatrics is presented as intellectually challenging and emotionally appealing (mean importance 3.06).

This is the largest cluster as it contains sixteen statements, including three top ten statements. The emphasis in this cluster is on geriatrics education being presented as a challenging and appealing specialization. A separate and compulsory geriatrics clerkship was regarded as an important prerequisite. Students have to be shown that geriatrics tries to seek out the most talented physicians. Moreover, the importance of geriatrics should be promoted by key figures in medical school such as the Dean, as well as by other medical specialists.

Cluster 4: There are senior-friendly role models (mean importance 3.0).

This cluster contains five statements, of which one can be found in the top 10. The statements consider the importance of positive role models, such as proud teachers but also residents. Nevertheless, physicians from other specializations will continue to show (often unconsciously) negative attitudes regarding the medical care for elderly patients, which they transfer to medical students. This might be counteracted by acknowledging this problem, paying attention from an early stage to professional identity development during medical school, and also by teacher professionalization on the job.

Cluster 5: Clear future professional perspectives are provided (mean importance 2.61).

None of the four statements in cluster 5 is found in the top ten. Nevertheless, all statements are about providing future professional perspectives - it must be clear to medical students who the key players in the field are, what the career perspectives, including financial rewards, are - and about helping students to find out what kind of doctor they want to become.

Cluster 6: (mean importance 2.63).

This is the smallest cluster containing two statements, neither one in the top ten. One of the students contributed the statement “making clear which treatment options there are”.

Like the previous statement, the other statement, “from individual physician to team player(s)”, could be perceived as a characteristic of geriatrics. However, the participant who presented this statement explained that this applied to all physicians, not just geriatricians: “all medical students should learn in medical school that a physician does not work alone, but always in cooperation with others, as a member of a team”. So these statements made it difficult to interpret the cluster and the researchers decided not to name this cluster. We still chose this grouping of six clusters, as the other five clusters did have a significant meaning and other cluster solutions grouped by the computer had less meaning*.*

#### Axes

The two axes in Fig. 1 can be seen as follows: statements above the y-axis concern the total medical curriculum, while the statements below the y-axis relate to dedicated geriatrics didactics. The statements to the right of the x-axis can be interpreted as tasks/challenges for curriculum designers and lecturers, whereas statements to the left of the x-axis can be seen as a clear perspective for medical students on the profession of geriatrician.

## Discussion

The main aim of this research was to find elements for a broad curriculum framework, which might raise medical students’ enthusiasm for the medical care of elderly patients.

The first element is: A patient centered medical curriculum. This element addresses not only the elderly patients, but teaches a more holistic state of mind for all medical problems. This element relates to an essential paradigm shift.

In the early twentieth century, medical education in the United States and Canada was revolutionarily reformed by the Flexner report of 1910. One of these reformations entailed the establishment of the biomedical model in medical training. Although in the last decades medical schools have shifted their focus more towards competence-based learning, and incorporated more or less psychosocial or behavioral science components in curricula, it is still characterized by a dualty, soma and psyche. The focus of clinical education remains on diagnosing and curing a disease and as a supplement, some attention for the individual patient, his context and care needs.

Medical students remain biomedical and disease focused and they do not appreciate education about psychological and social aspects [29].

This disease oriented model is not sufficient anymore in a time with a lot of patients with chronic diseases and multi-morbidity [30].

The statements in cluster 1, articulate a concept with a focus on the whole patient, the patient perspective and patient problems, less emphasis on the biomedical paradigm and more attention on care aspects. When the patient problems and the goals of the patient are the starting point of clinical decision-making, with the goal of restoring or remaining function, biomedical, psychological and social knowledge is needed. With the use of knowledge from all domains the dichotomy between soma and psyche, cure and care will disappear [31, 32].

The second and third element both emphasize geriatrics education.

The second element is: A curriculum that represents the patient population. That implies that at least 50% of the content and the patient exposure should be geriatrics related.

The third element is the largest cluster, containing 16 statements: Geriatrics should be presented as intellectually challenging and emotionally appealing. While the second cluster implies that geriatric content is integrated in the entire curriculum, the statements in the third cluster show that in addition to integrated geriatrics education, specific attention to the effectiveness of geriatrics education is necessary, for example at least by realizing a compulsory clerkship.

Although in the early twenty-first century medical schools in the United States, funded by the John A. Hartford and the Donald W. Reynold foundation, developed geriatric programs, there is still not enough interest in a career in geriatrics.

Moreover, many medical schools still do not include geriatric content in their curricula [33–36]. And in medical schools that do have curricula with geriatric content, it is often undervalued, with little time spent learning about geriatrics even though the majority of these future physicians will end up caring for frail and complex elderly patients [37].

Some of the statements in this cluster support the included studies in the review of Tullo that raise medical students attitudes towards older adults, like ‘elderly people as role model‘and ‘organizing attractive elements in geriatrics such as E health or games ‘. A separate compulsory geriatric clerkship is also supported in this cluster.

However, even in a medical school that offers geriatrics in all four years of education, including exposure to healthy older adults, like the examples of Tullo and senior mentor programs, the medical students’ perspective on geriatrics was quite negative [38]. In this qualitative study, medical students articulated a lack of intellectual stimulation, yet they were overwhelmed by the complexity of the geriatric patient. They were frustrated by the inability to cure and experienced the decline of patients as depressing. So, emotionally appealing education is not enough and the question is how to present the complex geriatric patient with multimorbidity as challenging and how to stimulate them intellectually.

To challenge the medical students, they should be really involved in the multidisciplinary treatment and follow-up of these patients inside and outside the hospital, i.e. in the home situation and the nursing home, so that they not only feel challenged by the complex problems, but also learn how to deal with this complexity and see the impact of small interventions on patient’s functioning and quality of life. When geriatric content is embedded in a more holistic and patient centered curriculum, according to cluster 1, geriatrics will not be perceived as a strange entity.

Some statements in this cluster offer ideas how to stimulate them intellectually, like ‘the scientific gap that is there to discover is a great challenge’ and ‘the visibility of the scientific challenge of aging’.

The fourth element is: there are senior friendly role models. Role models such as geriatricians and general practitioners, who take pride in their work, are essential to achieve that students really come to understand the joy of good care for the elderly patients and feel the motivation coming from the intellectual challenge and from being part of an improving care system.

One study in the United States regarding career choice for family medicine showed that significantly more medical students choose family medicine as a career if at least one family medicine faculty member is in a leadership position [39]. As long as the negative attitude prevails among professionals, it is important to address this explicitly. The statement “teacher professionalization on the job”, was explained as providing training to make supervisors of trainees aware of this subconscious attitude and how it affects the medical student.

The fifth element is about clear professional perspectives and reflects students’ need for a clear image of the work and career opportunities of geriatricians to enable them to make a solid career choice. Here, again, preclinical education and a compulsory clerkship are important. However, as a medical student you are mainly exposed to resident activities rather than the actual specialist activities. For this reason medical schools must also explicitly address career choice.

Summarizing, with an integrated approach that accommodates the elements from this concept map, with a curriculum whose underlying philosophy is based on patient-centeredness - in which the goals of the patient are central and biomedical, psychological and social approaches are integrated -, with geriatric content both integrated throughout the entire curriculum and also in a specific course and clerkship, with inspirational role models from elderly care medicine, geriatrics or other physicians delivering geriatric care, but also with key figures in the curriculum promoting the discipline and the importance and challenging aspects of the medical care for elderly people, and explicit attention to career choice and career prospects, we expect more medical students to choose a career in geriatrics and doctors in all specializations to be able to provide more appropriate care to elderly patients.

### Strengths and limitations

This is the first study in this field that makes use of concept mapping. In general, concept mapping has proven to generate valid and reliable results [24]. By choosing physicians, students and curriculum designers as participants, we were able to build a framework with the integrated opinions of these three important stakeholders. Although we only included eight participants -where Trochim advises a group size of 10 to 20 people to ensure a wide variety of opinions- we could nevertheless ensure a broad range of viewpoints due to the three different types of stakeholders.

In order to get a thorough interpretation of the map, the interpretation phase was done by the researchers, without the participants, as their time was limited. Nevertheless, involvement of the participants during this phase might have strengthened the validity of our findings. Also, involvement of the participants in this phase could have helped to interpret cluster 6. The concept mapping session is carried out four years ago. There has not been an important change the last four years regarding this subject.

### Recommendations

#### Research

Since this was the first concept map in this field of research, it should ideally be repeated and compared to see if all the dimensions are covered.

Each cluster should be further specified and operationalized in focus group studies.

More studies are necessary to see which teaching methods make medical students feel challenged.

and emotionally involved in the medical care of the complex geriatric patients instead of bored or frustrated.

Further research is needed to ascertain if a curriculum built according to our concept map will increase the interest of medical students for a career in geriatrics including their attitudes regarding the medical care for elderly patients.

#### Education

Each medical school can develop their elderly friendly curriculum, based on the clusters from this concept map, with the individual statements acting as cues.

“They can for instance use the same categories of stakeholders complemented with decanal representatives to further discuss the steps to be taken, how to overcome the potential barriers and develop a suitable program for that particular medical school.”

## Conclusion

The need for elderly-friendly medical education is evident. Although there are studies about promising interventions that raise medical students’ attitudes towards older adults, it is not enough to make a sufficient amount of medical students enthusiastic for the medical care of elderly patients or a career in geriatrics. Therefore a broader approach is necessary. In this study we used concept mapping, which resulted in the 5 main themes for a comprehensive curriculum change: 1. patient-centeredness as an underlying concept 2. a substantial amount of geriatrics integrated in the curriculum, 3. geriatrics, also in separate education or at least a clerkship, presented as intellectually challenging and emotionally appealing, 4. positive role models, and 5. a clear presentation of future professional perspectives. These themes require refinement in further discussion, but they provide leaders in medical education with a clear task.
